# Association between periodontitis and serum c-reactive protein levels

**DOI:** 10.4317/jced.57041

**Published:** 2020-09-01

**Authors:** Rafael-Paschoal Esteves-Lima, Christian-Santiago Reis, Francisco Santirocchi-Júnior, Lucas-Guimarães Abreu, Fernando-Oliveira Costa

**Affiliations:** 1 PhD in Periodontics. Faculty of Dentistry of Federal University of Minas Gerais, Av, Antônio Carlos, 6627, Belo Horizonte (MG), 31270-901, Brazil; 2 Graduate in Dentistry. Faculty of Dentistry of Federal University of Minas Gerais, Av, Antônio Carlos, 6627, Belo Horizonte (MG), 31270-901, Brazil; 3 PhD in Pediatric Dentistry. Faculty of Dentistry of Federal University of Minas Gerais, Av, Antônio Carlos, 6627, Belo Horizonte (MG), 31270-901, Brazil; 4 PhD in Epidemiology. Faculty of Dentistry of Federal University of Minas Gerais, Av, Antônio Carlos, 6627, Belo Horizonte (MG), 31270-901, Brazil

## Abstract

**Background:**

Periodontal disease may be associated with more bacteria and consequent induction of a systemic inflammatory process, with changes in the levels of C-reactive protein (CRP). The purpose of this cross-sectional study was to evaluate the association between periodontitis and serum levels of C-reactive protein.

**Material and Methods:**

The sample comprised 100 individuals distributed into two groups according to serum levels of C-reactive protein: normal or altered. Social, biological and behavioral data were collected by means of a structured questionnaire. Additionally, a blood test was requested to measure C-reactive protein levels. CRP values less than 3 mg/l were considered normal. Periodontal clinical examination was conducted in each participant for analysis of probing depth, bleeding on probing and clinical attachment level. Descriptive statistics, univariate analysis and logistic regression were performed. Results were provided in odds ratio, confidence intervals and p values.

**Results:**

Individuals with altered C-reactive protein levels showed a higher prevalence of periodontitis than individuals with normal C-reactive protein levels (*p*=0.008). In the final logistic regression model, individuals with periodontitis were more likely to present altered C-reactive protein than individuals without periodontitis (OR=3.27, CI=1.42-7.52, *p*=0.005).

**Conclusions:**

The alteration of the C-reactive protein levels among individuals with a higher prevalence of periodontitis corroborates clinical evidence that periodontal infection has a systemic impact.

** Key words:**C-reactive protein, cytokines, periodontal diseases, periodontitis.

## Introduction

Periodontitis is a progressive inflammatory disease of bacterial etiology characterized by gum bleeding, increased probing depth, and attachment loss. The aggression caused by bacterial biofilm stimulates an immune and destructive response to periodontal tissues, leading to collagen destruction, apical migration of junctional epithelium and loss of alveolar bone (1).

C-reactive protein (CRP) is an acute phase protein considered a non-specific and highly sensitive inflammatory marker, produced by liver cells in response to various forms of injury to the body (2). Systemic infection, trauma and hypoxia are associated with an increased production of CRP. Moreover, inflammatory cytokines, such as interleukin 6, interleukin 1 and tumor necrosis factor α can trigger the production of CRP by hepatocytes (3). Factors, such as smoking, obesity, diabetes and pregnancy have been associated with high serum levels of CRP (2).

The translocation of bacteria and bacterial products of oral cavity can induce a systemic inflammatory process, characterized by high levels of pro-inflammatory cytokines, including increased levels of CRP (4). CRP has also been considered a significant risk factor for many systemic diseases, such as cardiovascular disease and type 2 diabetes (3,5). In this scenario, the hypothesis that individuals with periodontitis present modified levels of CRP has been raised.

Although, the fact that periodontitis may be associated with changes in the levels of inflammatory markers makes the systemic impact of periodontal alterations relevant, few studies have assessed the relationship between periodontitis and altered levels of CRP. Scientific evidence on this relationship is still controversial and reduction in CRP levels is not always observed after periodontal therapy (6). Additionally, almost all studies have evaluated the relationship between periodontitis and CRP in individuals with some comorbidity, especially cardiovascular disease. Therefore, the objective of this study was to evaluate the association between periodontitis and serum levels of CRP.

## Material and Methods

-Sampling, setting and period of data collection 

The sample of this cross-sectional study was composed of 100 individuals attending the outpatient clinic of Periodontology of the Dental School of the Federal University of Minas Gerais, Belo Horizonte, Brazil. Individuals were randomly selected from September 2017 to December 2018.

Individuals who were 18 years or older and those presenting a minimum of 12 teeth were included. Individuals living with the human immunodeficiency virus, pregnant women, individuals who had undergone periodontal treatment within the last three months, individuals who had undergone anti-inflammatory or antimicrobial therapy within the last three months, those with diabetes, individuals with any contraindication to clinical periodontal examination and individuals reporting any systemic health condition were excluded.

The reporting of this manuscript conforms to The Strengthening the Reporting of Observational Studies in Epidemiology (STROBE) guidelines (7). Approval of the Ethics and Research Committee was obtained (CAAE 37241214.2.0000.5097). This study was conducted in accordance with the Helsinki Declaration. Participants were informed about the objectives and the methods of the study. Those who accepted to participate signed an informed consent form.

-Sociodemographic and behavioral characteristics

Participants’ data on sociodemographic and behavioral characteristics were collected by means of an interview during which a structured questionnaire was used. Data on sex, age, family income (number of wages of all members of the family who were economically active), schooling (number of years of studying) and smoking (smokers, non-smokers) (8) were collected.

-Periodontal clinical examination

Clinical examination was carried out in four sites (mesial, buccal, distal and lingual) of all teeth for analysis of the following periodontal parameters for periodontal diagnosis: probing depth (PD), bleeding on probing (BOP) and clinical attachment level (CAL). During clinical examination, a manual periodontal probe (UNC-15, Hu-Friedy®) was used.

Three examiners (F.S.J., C.S.R. and R.P.E.L.), previously trained and calibrated, conducted the examination of the participants. Values of inter and intra-examiner agreement assessed by the interclass correlation coefficient were above 0.86 for PD and CAL.

Teeth with changes in gingival morphology, teeth with extensive caries lesions, those with poor restorative procedures and teeth, for which the determination of the limit between the enamel and cementum was unfeasible, were excluded (9).

-Definition of periodontitis and assessment of the prevalence

The criteria for periodontitis definition was the presence of ≥4 teeth having ≥1 sites with PD ≥4 mm and CAL ≥3 mm associated with BOP (2).

-Medical data

A blood test of each participant was ordered for quantification of CRP. The collection of blood samples and the analysis of the samples of all individuals were carried out providing a period of eight hours of fasting. CRP values less than 3 mg/L were considered normal. Values greater than or equal to 3 mg/L were considered altered (10). Thus, participants were divided into two groups, according to the serum levels of CRP: individuals with normal CRP and individuals with altered CRP.

Weight and height were measured for body mass index (BMI) calculation. Participants could be assigned to the following subgroups according to their BMI: underweight (BMI below 18.5), normal weight (BMI between 18.5 and 24.9), overweight (BMI between 25 and 29.9) and obese (BMI above 29.9).

-Statistical analyses

Data analyzis was performed using the Statistical Package for the Social Sciences (SPSS Inc., version 23.0, Armonk, USA). Descriptive analyzis was conducted. The Mann-Whitney test was used to compare individuals with normal CRP and those with altered CRP with respect to sociodemographic variables (sex, age, family income and schooling) as well as smoking and BMI. The Mann-Whitney test was also used to compare individuals with normal CRP and those with altered CRP with respect to periodontitis (percentage of individuals with or without periodontitis). Finally, a regression analysis evaluating the association between periodontitis and CRP alterations controlling for confounding variables (age, smoking and BMI) was carried out. In all analyzis, results with a probability lower than 5% were considered significant (*P*<0.05).

## Results

Comparisons between the groups with normal and altered CRP levels regarding the sociodemographic variables (sex, age, family income and schooling) as well as smoking, BMI and periodontitis are presented in Table 1. No significant difference between groups regarding sex, age, family income, schooling and smoking was observed. Individuals with altered CRP presented a mean BMI of 33.0, while individuals with normal CRP presented a mean BMI of 26.0. A significant difference between groups was observed (*P*<0.001). The prevalence of periodontitis among individuals with altered CRP (66.7%) was significantly higher than among individuals with normal CRP (37.9%) (*P*=0.008).

**Table 1:**
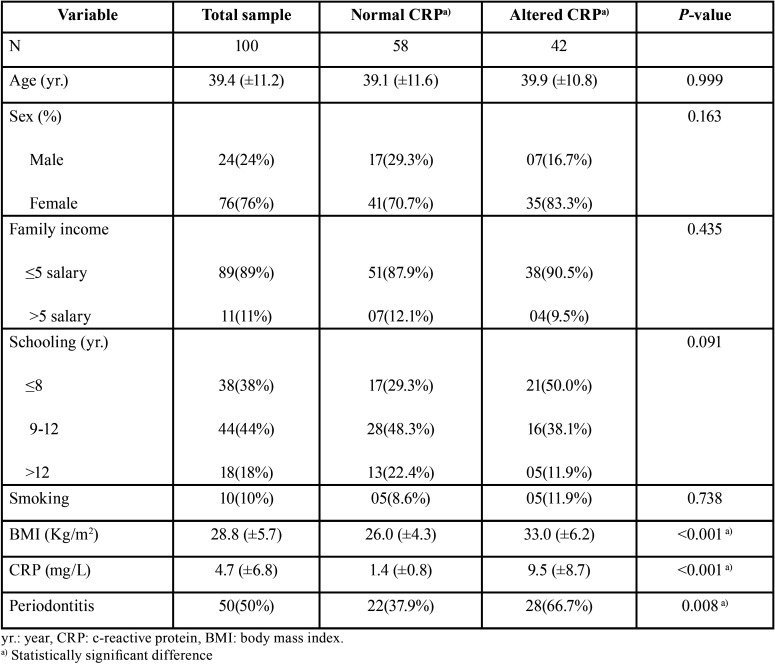
Comparisons between individuals with normal and altered CRP with respect to independent variables.

The regression analyzis demonstrated that the levels of CRP among individuals with periodontitis were 1.72 times higher than the levels of CRP among individuals without periodontitis (confidence interval=1.02 – 2.93; *P*=0.042), regardless of the influence of the cofounding variables age, smoking and BMI (Table 2). The analyzis also showed that the levels of CRP among obese individuals were 3.48 times higher than the levels of CRP among underweight individuals/individuals with normal weight (confidence interval=1.56 – 7.75; *P*=0.002).

**Table 2:**
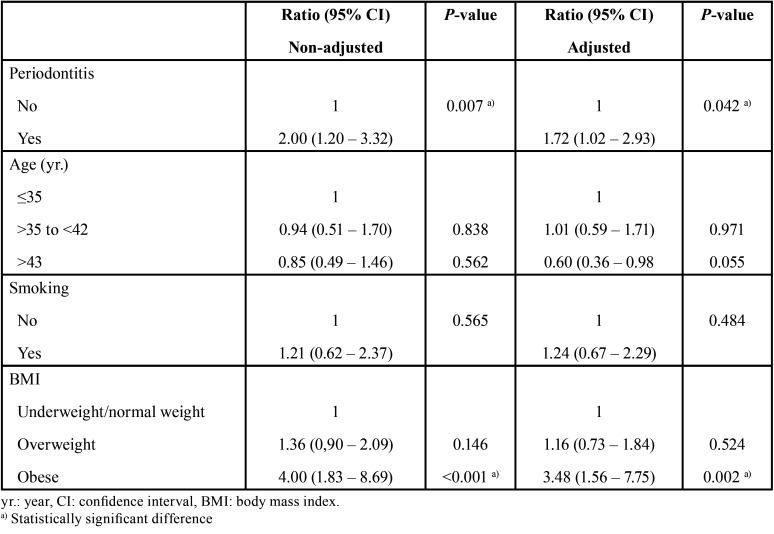
Regression analysis evaluating the association of periodontitis and CRP alteration, controlling for confounding variables (age, smoking and body mass index.

## Discussion

CRP, a marker for acute inflammation, is produced by the liver as a result of various types of injuries, including infectious illnesses. Inflammatory mediators arising from periodontitis may stimulate hepatocytes to produce CRP. Among these mediators, interleukin-1, interleukin-6 and tumor necrosis factor alpha are particularly involved in this process (2). In this sense, periodontal infection may lead to systemic inflammation with a significant increase in CRP levels. The results of the present study showed that individuals with periodontitis presented a significantly higher level of CRP in comparison with individuals without periodontitis. These results allow one to accept the hypothesis that periodontitis is associated with a higher production of this nonspecific inflammatory marker with far-reaching effects on the individual affected by periodontitis. In one previous study (11), the authors evaluated how systemic inflammation mediated the association between periodontitis and glycemic status. The results of their study showed that CRP, white blood cell count and platelet-lymphocyte ratio mediated 8%, 13% and 16% of the association between severe periodontitis and diabetes. Other studies have described findings similar to our results on the association between periodontitis and serum levels of CRP (12-16). One study (17), for instance, demonstrated that the presence of *Porphyromonas gingivalis* increases CRP levels in nearly 20%. It should be emphasized that almost all studies analyzing the association between periodontitis and serum CRP levels have evaluated individuals with any systemic changes, such as cardiovascular disease, diabetes or rheumatoid arthritis. The presence of systemic changes can be a confounding factor in assessing the impact of periodontitis on CRP levels. In our study, only individuals without any systemic alteration participated. The exclusion of individuals who had reported the presence of any systemic alteration during an interview strengthens the present study. However, it is possible that some unknown systemic changes may be present in some individuals in the sample, which is a limitation.

Additionally, the severity of periodontitis may have a direct impact on the levels of CRP. One study (18) demonstrated that CRP levels increase with the severity of periodontitis. Cases of very progressive and extensive periodontitis are associated with higher CRP levels (12,13,15,19-21). As regards periodontal therapy, however, intervention studies show discrepancies whether periodontal treatment has an impact on the reduction of serum CRP levels (14,15,21-23).

In addition to the periodontal infection, other factors may be associated with the increased production of CRP. This fact may explain why some individuals without periodontitis presented significant changes in CRP levels. Factors such as age, smoking and obesity have been associated with alterations of the levels of CRP (24). Older individuals, smokers and individuals with obesity may have increased levels of this marker. This is the reason why regression analyzis assessing the relationship between periodontitis and CRP levels, controlling for these confounders has been conducted herein.

In this study, individuals with normal CRP and altered CRP were roughly equal in age, and the mean age of the sample was not high. CRP concentration increases with age, probably due to the increased incidence of subclinical pathological conditions. Age is a highly relevant factor that should be taken into account when CRP is assessed. This result diverges from the fact that a higher age is associated with increased levels of CRP (24). On the other hand, the prevalence and severity of periodontitis may also increase with age (25). Therefore, age is a confounding factor that should be evaluated in studies in which the association between periodontitis and serum CRP levels is assessed. The similarity between groups of individuals with normal and altered CRP serum levels regarding age is a strong point of the present study, favoring the analysis of the relationship between periodontitis and CRP.

Herein, the regression analyzis demonstrated that obese individuals presented significantly higher levels of CRP in comparison with underweight individuals/normal weight individuals. The scientific literature has shown that obesity is associated with high levels of CRP because the secretion of inflammatory cytokines is increased in individuals affected by obesity (26). Obesity has also been associated with periodontitis. The secretion of cytokines by the adipose tissue leads to a more exacerbated inflammatory response with a strong impact on the pathogenesis of periodontitis (27).

Smoking has been associated with high levels of CRP (24). Smoking is also a risk factor for periodontitis. Tobacco use plays an important role in the pathogenesis of inflammatory periodontal disease. Smokers present greater prevalence and severity of periodontitis (2). Herein, no significant difference between groups regarding the number of smokers was observed. Moreover, the regression analyzis also demonstrated that the levels of CRP among smokers were not significantly different in comparison with the levels of the non-smokers. The low number of individuals who were smokers and participated in this study may explain the similarity between groups regarding the smoking habit. In addition, CRP levels appear to be higher in women and in individuals with low socioeconomic status (28,29). Importantly, in this study, the groups of individuals with normal and altered levels of CPR were similar in relation to sex, family income and schooling.

The definition of the criteria for periodontitis diagnosis is an important methodological step in studies in which the association between periodontal disease and levels of inflammatory markers is evaluated. The criteria used for the diagnosis of periodontitis may have a great impact on the prevalence of the disease (30). The definition of periodontitis should not underestimate or overestimate the disease. The characteristics of the periodontal condition of the sample should also be considered. Individuals with differences in the extension and severity of the periodontal disease may present different systemic behaviors. This fact should be considered when interpreting the results and conclusions of the different studies. Additionally, a thorough periodontal examination is necessary to establish an appropriate diagnosis. In the present study, a complete periodontal clinical examination was performed to evaluate the periodontal parameters and a robust diagnostic criterion was used to assess the systemic impact of periodontitis.

In this study, individuals with periodontitis presented higher levels of CRP in comparison with individuals without periodontitis. This positive association reinforces the theory that periodontitis has a significant influence on the levels of inflammatory biomarkers, suggesting that periodontal infection can lead to a systemic impact, favoring the development and aggravation of other pathologies. However, additional studies, in particular intervention and longitudinal studies, with special attention to confounding factors, are needed to further assess the association between periodontitis and serum levels of CRP.
